# The Immunobiological Agents for Treatment of Antiglomerular Basement Membrane Disease

**DOI:** 10.3390/medicina59112014

**Published:** 2023-11-16

**Authors:** Marina Yamashita, Mamiko Takayasu, Hiroshi Maruyama, Kouichi Hirayama

**Affiliations:** Department of Nephrology, Tokyo Medical University Ibaraki Medical Center, Ami 300-0395, Ibaraki, Japan; yamashit@tokyo-med.ac.jp (M.Y.); t-mamiko@tokyo-med.ac.jp (M.T.); maruhiro@tokyo-med.ac.jp (H.M.)

**Keywords:** anti-glomerular basement membrane disease, rituximab, belimumab, tumor necrosis factor-alpha, abatacept, tocilizumab, eclizumab

## Abstract

Combination therapy with glucocorticoids, cyclophosphamide, and plasmapheresis is recommended as the standard treatment for anti-glomerular basement membrane (anti-GBM) disease, but the prognosis of this disease remains poor. Several immunobiological agents have been administered or are expected to be useful for anti-GBM disease in light of refractory disease or the standard treatments’ tolerability. Many data regarding the use of biologic agents for anti-GBM disease have accumulated, verifying the effectiveness and potential of biologic agents as a new treatment option for anti-GBM disease. Tumor necrosis factor (TNF) inhibitors were shown to be useful in animal studies, but these agents have no clinical use and were even shown to induce anti-GBM disease in several cases. Although the efficacy of the TNF-receptor antagonist has been observed in animal models, there are no published case reports of its clinical use. There are also no published reports of animal or clinical studies of anti-B-cell-activating factor, which is a member of the TNF family of agents. Anti-interleukin (IL)-6 antibodies have been demonstrated to have no effect on or to exacerbate nephritis in animal models. Anti-C5 inhibitor was observed to be useful in a few anti-GBM disease cases. Among the several immunobiological agents, only rituximab has been demonstrated to be useful in refractory or poor-tolerance patients or small uncontrolled studies. Rituximab is usually used in combination with steroids and plasma exchange and is used primarily as an alternative to cyclophosphamide, but there is insufficient evidence regarding the efficacy of rituximab for anti-GBM disease, and thus, randomized controlled studies are required.

## 1. Introduction

Anti-glomerular basement membrane (anti-GBM) disease is a rare autoimmune disorder characterized by rapidly progressive glomerulonephritis (RPGN) with diffuse crescentic formation on renal biopsy, and it is a well-characterized cause of glomerulonephritis [1]. Anti-GBM disease was defined as the presence of serum autoantibodies to the noncollagenous domain of the alpha 3 chain of type IV collagen or a linear binding of immunoglobulin G (IgG) to glomerular capillary walls as detected with direct immunofluorescence in patients with RPGN [2]. More recently, anti-GBM disease has been categorized as one of the types of vasculitis [3]. A group of diseases with clinical manifestations that differ from those of typical anti-GBM disease has also been identified: (i) atypical anti-GBM disease (mild renal lesion type, IgG4 anti-GBM antibody type), (ii) membranous nephropathy-complicated type, (iii) anti-neutrophil cytoplasmic antibody (ANCA)-complicated type, and (iv) post-renal transplantation-onset type (Alport syndrome), which has been proposed as a special disease type [4,5]. A further accumulation of and investigations of patients with these disease types are necessary.

As the pathogenesis of anti-GBM disease became clear, treatment regimens were designed to remove the circulating pathogenic anti-GBM antibodies with therapeutic plasma exchange, to attenuate the pathogenic antibody-mediated glomerular inflammatory responses with the administration of corticosteroids, and to suppress the further production of these pathogenic antibodies with the use of immunosuppressive agents. In the Kidney Disease Improving Global Outcomes (KDIGO) guidelines for the management of glomerular diseases [6], combination therapy with glucocorticoids, cyclophosphamide, and plasmapheresis is recommended. Although effective treatments to improve renal function have been reported, the prognosis for patients with anti-GBM disease is poor (Supplementary File Table S1) [7,8,9,10,11,12,13,14,15,16,17,18,19,20,21,22,23,24,25,26,27,28,29,30,31].

As new treatment options, several immunobiological agents have been tried or are expected to be useful for anti-GBM disease, as such agents have been demonstrated to be effective in other kidney or rheumatic diseases and/or have been clinically applied. For example, the efficacy of rituximab has been demonstrated for anti-neutrophil cytoplasmic antibody (ANCA)-associated vasculitis (AAV), which is classified as ‘small vasculitis’ (as is anti-GBM disease) [32,33]. In addition to rituximab, anti-BAFF (B cell activating factor), which is a member of the tumor necrosis factor (TNF) family of BAFF/B lymphocyte stimulator (BLyS) agents, is effective for the treatment of systemic lupus erythematosus (SLE) [34], and TNF-alpha inhibitors were useful as the first immunobiological agent used to treat rheumatoid arthritis [35].

In this review, we have gathered and analyzed the existing literature on immunobiological agents in the treatment of anti-GBM disease, and we describe the agents’ usefulness and potential as new treatment options for anti-GBM disease.

## 2. Review Methods

We searched the literature available up to December 31, 2022 in the following electronic databases: PubMed/MEDLINE, EMBASE, and Web of Science: Science Citation Index Expanded. The key words that were used included ‘antiglomerular basement membrane disease’ or ‘Goodpasture’s syndrome’ and (‘rituximab’ or ‘CD20’), (‘belimumab’, ‘atacicept’, ‘tabalumab’, ‘blisibimod’, ‘BAFF’, or ‘BLyS’), (‘infliximab’, ‘adalimumab’, ‘golimumab’, ‘certolizumab’, or ‘anti-TNF-α’), (‘etanercept’, or ‘TNF-α inhibitor’), (‘abatacept’, ‘CD28’, ‘CD80’, ‘CD86’, or ‘CTLA-4’), (‘tocilizumab’, ‘sarilumab’, or ‘IL-6’), or (‘eculizumab’, or ‘ravulizumab’) (Supplementary File Table S2).

We detected 67 reports associated with rituximab, and among them, seven were excluded as they were in a language other than English. Twelve other reports were excluded because of the publication type (e.g., review). Among the remaining 48 reports, five were excluded because the subjects had different diseases: membranous nephropathy (*n* = 2) and AAV (*n* = 3). Two case reports of patients treated without rituximab were excluded. A final total of 34 case reports (Supplementary File Table S3) [36,37,38,39,40,41,42,43,44,45,46,47,48,49,50,51,52,53,54,55,56,57,58,59,60,61,62,63,64,65,66,67,68,69] and seven cohort studies [28,70,71,72,73,74,75] (Supplementary File Table S4) were obtained and qualified.

Regarding BAFF (BLyS), with the exception of a single review article that concerned other vasculitis diseases treated with belimumab, there was no report of anti-GBM disease treated with anti-BAFF monoclonal antibody. However, we identified an investigation of serum BAFF levels in patients with anti-GBM disease [76].

In the literature associated with anti-TNF-α monoclonal antibodies, only one study that used an animal model [77] and one study of adalimumab were detected [78]. We found no report of anti-GBM disease together with infliximab, golimumab, or certolizumab. In the literature concerning TNF-α inhibitors, only one report of etanercept was detected [63]. No clinical study of anti-GBM disease treated with anti-TNF-α monoclonal antibodies or TNF-α inhibitors was identified.

Regarding abatacept, CD28, CD80, CD86, or cytotoxic T-lymphocyte-associated protein 4 (CTLA)-4, we detected 12 reports, and among them, we excluded the single report in a language other than English, three review reports, and one animal-model study of other molecules (Fc receptor). Of the remaining seven reports, three studies involved the use of abatacept or CTLA-4 monoclonal antibodies [79,80,81], and all three studies were of animal models. No clinical study of anti-GBM disease treated with abatacept was identified.

We detected 27 reports of the use of interleukin (IL)-6 in anti-GBM disease, and among them, we excluded two reports in a language other than English. Twenty reports were animal models of anti-GBM disease, and 17 of those 20 studies were excluded because they included no use of anti-IL-6 monoclonal antibodies. Of the remaining five case reports excluding animal studies, we excluded three because they concerned diseases other than anti-GBM disease; two reports were of patients with pauci-immune crescentic glomerulonephritis, and the other was of a patient with Castleman disease. Among the two remaining anti-GBM disease papers, neither used anti-IL-6 monoclonal antibodies. A final total of three papers in which anti-IL-6 monoclonal antibodies were used in animal models [82,83,84] were available.

In the anti-C5 monoclonal antibody literature, six reports associated with eclizumab and no report associated with ravulizumab were detected. Among the six eclizumab reports, two were in a language other than English, and the subjects in two other reports had different diseases: C3 nephropathy and hemolytic uremic syndrome (HUS). Only two case reports of patients with anti-GBM disease treated with eclizumab [85,86] were obtained.

## 3. Results

### 3.1. B Cell Co-Receptors (CD20)

Autoantibodies produced by B cells are thought to play a role in the development of several autoimmune diseases. On the surface of B cells, B cell receptor, B cell co-receptors (the CD21:CD19:CD81 complex), and CD20 are expressed [87]. The anti-CD20 monoclonal antibody transduces a signal that induces B cell apoptosis and depletes B cells for several months [88]. Although CD20 is not expressed on antibody-producing plasma cells, these cells’ B cell precursors are targeted by anti-CD20 monoclonal antibody, resulting in a substantial reduction in the short-lived plasma cell population [88].

Rituximab was originally developed to treat B cell lymphoma [89], but it has also been demonstrated to be effective in several autoimmune diseases, such as AAV [31,32], refractory SLE [90], SLE-associated antiphospholipid syndrome [91], and acquired thrombotic thrombocytopenic purpura [92]. Our group analyzed the cases of 75 patients with anti-GBM disease who were treated with rituximab (37 patients in 34 case reports [36,37,38,39,40,41,42,43,44,45,46,47,48,49,50,51,52,53,54,55,56,57,58,59,60,61,62,63,64,65,66,67,68,69] and 38 patients in seven cohort studies [28,70,71,72,73,74,75]), but these cohort studies also included a selection of patients with anti-GBM disease who were treated with rituximab from among different disease subjects, for example, 11 cases treated with rituximab among 119 anti-GBM disease cases [28], one anti-GBM disease case treated with rituximab among 60 cases of crescentic glomerulonephritis [72], and two cases treated with rituximab among 12 cases of anti-GBM disease and membranous nephropathy [73].

The characteristics and clinical features of the final total of patients are summarized in Table 1. The patients (*n* = 63) were 34 males and 29 females with a male predominance ratio of 1.17. The mean age was 43.7 years (range 2–91 years), and 14 of the 63 patients (22.2%) were elderly (>65 years). Kidney involvement was observed in 61 of 62 patients (98.4%), but pulmonary involvement was identified in 26 of 53 (49.1%) patients. The mean serum creatinine level at the initial presentation was 6.95 mg/dL (range, 0.34–46.6 mg/dL; *n* = 59). In addition, 30 of 49 patients (61.2%) needed to undergo hemodialysis before the initial treatment. A renal biopsy was performed in 51 of 57 (89.5%) patients, and various degrees of necrotizing crescentic glomerulonephritis were observed. Twenty-eight of 39 patients (71.8%) had >50% crescentic glomeruli, and the mean percentage of glomeruli showing crescent formation was 67.5% (range 3–100%; *n* = 39). Serum anti-GBM antibody was detected in 56 of 61 patients (91.8%), and the mean titer of serum anti-GBM antibody was 267.8 U/mL (range 0–3060 U/mL; *n* = 47).

An oral corticosteroid was used in 62 of 64 patients (96.9%), and intravenous corticosteroid therapy was used in 48 of 56 patients (85.7%). Compared to corticosteroid therapy, the rate of cyclophosphamide use was lower; oral cyclophosphamide was administered in 25 of 58 patients (43.1%), and intravenous cyclophosphamide was used in 18 of 54 patients (33.3%). Plasma exchange was performed in 54 of 62 patients (87.1%).

Rituximab was used as the remission-induction treatment in all 63 cases (Table 2), and it was used as the initial treatment in 11 of 61 cases (18.0%). The reasons for the use of rituximab were resistance to standard treatments in 19 of 49 cases (38.8%), relapse of anti-GBM disease in eight cases (16.3%), and poor tolerability of cyclophosphamide in 20 cases (40.8%). The dose of rituximab varied depending on the patient, but 375 mg/m^2^ and 1000 mg were each more common (375 mg/m^2^ in 35 cases and 1000 mg in 10 cases), and the mean number of times of rituximab administration was 3.2 times (range 1–8; *n* = 54). There was no difference in the remission rate or adverse effect rate described below due to the difference in administration method.

The mean follow-up period was 19.8 months (range 2–93 months; *n* = 55). Only one patient had died due to pulmonary infection [55], and 26 of 64 patients (40.6%) were ongoing end-stage renal disease with maintenance dialysis therapy (Table 2). The mean serum creatinine level at the last follow-up was 1.83 mg/dL (range 0.54–4.52 mg/dL; *n* = 18), and 42 of 44 patients (95.5%) had a negative anti-GBM antibody result at their last follow-up. The main adverse event was infection, which was reported in six out of 37 cases in the case report (one patient with candidiasis [37], one aspergillosis [52], one cytomegalovirus [54], one bacterial infection [55], two pneumocystis pneumonia [59,64]) and three out of 38 in the cohort study (one patient with bacterial infection [71], two candidiasis [70,71]). However, there is a possibility that the infection is due to other concomitant medications, including glucocorticoids and cyclophosphamide, so it cannot be said that rituximab is the only cause. In fact, similar infectious complications have been reported with combination therapy with glucocorticoids, cyclophosphamide, and plasmapheresis. Although there are a few cases of other adverse events, the following have been reported: posterior reversible encephalopathy syndrome in three patients and progressive leukoencephalopathy in two patients.

In summary, rituximab has been shown to be useful in many case reports or small uncontrolled studies. Rituximab is used in the cases of patients who are resistant to standard treatments or have low tolerance to standard treatments. Rituximab has often been used in combination with steroids and plasma exchange, and it is used primarily as an alternative to cyclophosphamide. However, the amount of rituximab used, the dosing interval, and the number of doses vary, and there is no established standard, thus, requiring further research. Randomized controlled studies are also necessary to determine the effectiveness of rituximab, as no degree of superiority or inferiority has been confirmed compared to standard treatments.

### 3.2. B Cell Activating Factor/B Lymphocyte Stimulator

BAFF (also known as BLyS) is a member of the TNF superfamily 13B (TNFSF13B) of proteins that regulate immune responses [93]. BAFF binds to three receptors that are present on several immune cell types: (i) BAFF receptor (BAFF-R; also known as BR3 and TNF-receptor superfamily (TNFRSF)-13C), (ii) transmembrane activator and calcium modulator and cyclophilin ligand interactor (TACI; also known as TNFRSF13B), and (iii) B cell maturation antigen (BCMA; also known as TNFRSF17) [93]. Mice that are transgenic for BAFF have greatly increased numbers of mature B cells and effector T cells, and they develop autoimmunity [94], whereas BAFF-deficient mice lack mature B cells [95]. It was also reported that, although under normal BAFF concentrations the non-self-reactive B cells survived and the autoreactive B cells were deleted, a higher BAFF concentration contributed to the survival of autoreactive B cells and elevated the production of autoantibodies [93].

Four biologic drugs have been developed in an attempt to block the BAFF-BAFF receptors pathway: belimumab, atacicept, tabalumab, and blisibimod. Belimumab is a human monoclonal antibody that antagonizes the effect of BAFF by binding to the soluble form of BAFF [96]. Atacicept is a TACI-Fc fusion protein that binds to and blocks the receptor for BAFF [97]. Tabalumab and blisibimod both also block the active forms of BAFF; tabalumab is a human monoclonal antibody, and blisibimod is a fusion polypeptide protein [98].

In several studies investigating the use of belimumab in patients with SLE, it was demonstrated that the SLE Responder Index (SRI) response rate in the patients treated with belimumab was significantly higher than that in the placebo-treated patients [99]. The use of belimumab for patients with other rheumatic diseases has been investigated, but the efficacy of belimumab in those diseases was limited: a phase II trial of belimumab for rheumatoid arthritis (RA) [100], a phase II open-label clinical trial of belimumab for Sjögren’s syndrome [101], and a randomized controlled trial (RCT) of belimumab as a maintenance therapy in AAV [102]. The observed utility of other anti-BAFF agents (atacicept, tabalumab, and blisibimod) for kidney or rheumatic diseases is even more limited and has not yet reached clinical application. In phase II/III trials of patients with RA, atacicept did not improve the American College of Rheumatology response rates [103,104]. Two phase II/III trials of atacicept for SLE were terminated early due to safety concerns [105,106]. Tabalumab and blisibimod both exhibited disappointing efficacy for SLE in recent phase III clinical trials [107,108,109].

Our searches related to BAFF and anti-GBM disease identified only one paper on serum BAFF levels in patients with anti-GBM disease, but we found no animal studies involving anti-BAFF agents (belimumab, atacicept, tabalumab, or blisibimod) and no clinical studies (RCT, cohort study, or case report) involving these drugs. In the single paper on serum BAFF levels in patients with anti-GBM disease, the patients’ serum BAFF levels were significantly higher than those of healthy controls, and the patients’ levels were associated with the percentage of glomeruli with crescents [76]. The efficacy of anti-BAFF monoclonal antibodies for the treatment of patients with anti-GBM disease remains to be clarified.

### 3.3. Tumor Necrosis Factor (TNF)-α

TNF-α is produced as membrane-bound TNF-α, a precursor protein with a molecular weight of 25 kDa [110]. The extracellular carboxyl-terminal domain of TNF-α is cleaved by TNF-α-converting enzyme (TACE) to form 17 kDa soluble TNF-α [110]. TNF-α is involved in the human body’s defense against infection and anti-tumor effects through the expression of cell-adhesion molecules, the induction of apoptosis, the production of inflammatory mediators, and the enhancement of antibody production by plasma cells [110]. However, overexpression of TNF-α can result in autoimmune diseases, such as RA and psoriasis [110]. Anti-TNF-α monoclonal antibodies (as potent, multifunctional, monoclonal antibodies) not only play an important role in the immune system’s homeostatic function but also exert excellent anti-inflammatory effects in various autoimmune diseases [111].

Infliximab is a genetically constructed IgG1 mouse–human chimeric (75% human-derived and 25% mouse-derived amino acids) monoclonal antibody that binds both the soluble subunit and the membrane-bound precursor of TNF-α [112]. Adalimumab [113] and golimumab [114] are fully human IgG1 monoclonal anti-TNF-α antibodies. Certolizumab pegol is a pegylated Fc-free Fab’ fragment of a humanized anti-TNF-α monoclonal antibody [115]. These anti-TNF-α monoclonal antibodies have been demonstrated to be effective in RA [116], juvenile idiopathic arthritis [117], psoriatic arthritis [118], ankylosing spondylitis [119], and inflammatory bowel disease [120], and each has been clinically applied.

In a mouse model of anti-GBM disease, TNF-α knockout mice exhibited a reduction in the severity of crescent formation [121]. In an experimental study of crescentic glomerulonephritis, the administration of anti-TNF-α monoclonal antibody reduced glomerular inflammation, crescent formation, and tubulointerstitial scarring with preservation of renal function [77]. Our literature search revealed no clinical studies (RCT, cohort study, or case report) involving anti-TNF-α monoclonal antibodies (infliximab, adalimumab, golimumab, or certolizumab pegol). However, a patient with RA in whom anti-GBM disease developed 4 weeks after the administration of adalimumab was reported [78].

In summary, although animal studies have indicated that anti-TNF-α monoclonal antibodies may be useful in the treatment of anti-GBM disease, there are no reports of the use of these antibodies in clinical studies. On the other hand, there is the above-cited report that anti-GBM disease developed after the administration of adalimumab, and caution should, thus, be exercised when using anti-TNF-α monoclonal antibodies for anti-GBM disease.

### 3.4. TNF-α Receptor

There are two types of cell-surface TNF receptors: p55 TNF receptor-I (TNFRSF1A) and p75 TNF receptor-II (TNFRSF1B) [122]. A soluble, truncated membrane TNF receptor that consists of only an extracellular ligand-binding domain is thought to be involved in the regulation of TNF activity [122]. Etanercept is a recombinant dimeric fusion protein that consists of the extracellular ligand-binding portion of the human p75 TNF receptor linked to the Fc portion of human IgG1 [123]. Etanercept has been demonstrated to be effective against RA [124], juvenile idiopathic arthritis [117], psoriatic arthritis [118], and ankylosing spondylitis [119], and it has been applied clinically.

Regarding experimental anti-GBM disease, our literature search turned up no report of etanercept. There was also no report of etanercept used for patients with anti-GBM disease. On the other hand, anti-GBM disease was induced with etanercept in one of the patients who had been treated with rituximab [63]. That patient had been treated for psoriatic arthritis with weekly etanercept for the past 12 months but subsequently developed anti-GBM disease with hematuria and deteriorated renal function.

In summary, there have been no reports of the use of etanercept for anti-GBM disease in animal experiments or clinical studies, but there is a report that anti-GBM disease occurred with etanercept use, and caution should, thus, be exercised when using this agent for anti-GBM disease.

### 3.5. Co-Stimulatory Molecules of T Cells

The activation of T cells requires two distinct signals: the first is an antigen-specific interaction between the T cell receptor and the nominal antigen presented in the context of the major histocompatibility complex (MHC) on the surface of an antigen-presenting cell, and the second signal may be provided through a number of potential co-stimulatory molecules of which CD28 may be the most important [125]. Co-stimulation is especially important for the initial T cell response, and its effects are mediated by promoting the cells’ proliferation and survival. CD28 is present on most T cells, and it binds to both CD80 and CD86, which are present on antigen-presenting cells, including dendritic cells, B cells, and macrophages [125]. Cytotoxic T lymphocyte-associated antigen (CTLA)-4 (CD152), which is upregulated on T cells following their activation, also interacts with CD80 and CD86, providing an important mechanism for regulating T cell function [126].

Abatacept is a recombinant fusion protein comprised of the extracellular domain of human CTLA4 and a fragment of the Fc domain of human IgG1. It competes with CD28 for CD80 and CD86 binding, and it can, thus, be used to selectively modulate T cell activation [127]. Abatacept has been demonstrated to be effective against RA [117], juvenile idiopathic arthritis [128], and psoriatic arthritis [129], and it has been clinically applied.

Anti-GBM disease induction with anti-GBM antiserum was nearly prevented in CD28-deficient mice [130]. In an experimental anti-GBM disease model in CD88-deficient mice, the degree of glomerular lesions was exacerbated, whereas the corresponding degree in CD80-deficient mice was attenuated [131]. In the glomeruli of mice with anti-GBM disease, both CD80 and CD86 molecules were upregulated, and the administration of anti-CD80/86 monoclonal antibodies attenuated the glomerular accumulation of CD4^+^ T cells and macrophages, crescent formation, and proteinuria [132]. Thus, co-stimulation may be important in the development of anti-GBM disease. In an experimental anti-GBM disease model in Wistar–Kyoto rats, the administration of the fusion protein human CTLA4-immunoglobulin (which binds to CD80 and CD86) [79] and the mutant CTLA4-immunoglobulin (which binds only to CD80) [80] reduced disease severity. The fusion protein human CTLA4-immunoglobulin also attenuated glomerular lesions in an experimental mouse anti-GBM disease model [81]. However, our literature search identified no report of abatacept for patients with anti-GBM disease.

In summary, although animal studies have indicated that abatacept may be useful in the treatment of anti-GBM disease, there are no reports of this use in clinical studies. Further investigation is required regarding the efficacy of anti-CTLA-4 antibodies.

### 3.6. Interleukin-6

IL-6 is a cytokine that differentiates activated B cells into antibody-producing cells, and IL-6 has been shown to have various biological activities (e.g., acute-phase reactive protein production, angiogenesis, neutrophil activation and migration, and immunocompetent cell differentiation and activation) and to play a central role in inflammatory responses [133]. Increased IL-6 production is known to be involved in the pathogenesis of many autoimmune and inflammatory diseases [133].

Tocilizumab is a humanized, anti-human, IL-6 receptor monoclonal antibody; the complementarity-determining region of the variable region is a mouse-type, human, IL-6 receptor monoclonal antibody, and the residual part is human IgG1 [134]. Sarilumab is a fully human, anti-IL-6 receptor monoclonal antibody [135]. These agents bind to the IL-6 receptor with high affinity and inhibit signal transduction via IL-6, thereby suppressing excessive inflammatory reactions derived from IL-6. Anti-IL-6 receptor monoclonal antibodies have been demonstrated to be effective against RA [136], juvenile idiopathic arthritis [137], adult-onset Still’s disease [138], Takayasu arteritis [139], giant cell arteritis [140], Castleman disease [141], and Coronavirus disease 2019 (COVID-19) [142], and they have been clinically applied.

Increased IL-6 and IL-6 gene expression in glomeruli were observed in an animal model of anti-GBM disease [143]. In another anti-GBM animal model, the administration of rapamycin (sirolimus), which binds to the mammalian target of rapamycin (mTOR), inhibited cell proliferation signals and produced immunosuppressive effects, resulting in decreased proteinuria, reduced inflammatory cell infiltration, and decreased IL-6 expression, providing an improvement of nephritis [144]. In human anti-GBM disease, it has been demonstrated that the serum or urine IL-6 level is elevated, and the glomerular IL-6 expression is enhanced [82,145]. However, a continuous infusion of IL-6 to a rat model of anti-GBM disease reduced renal inflammation and preserved renal function [146]. Anti-IL-6 antibody had no significant effect on histological changes, renal function, or proteinuria in an anti-GBM disease model [82]. A pre-emptive treatment of mice with anti-GBM disease using anti-IL-6 receptor or anti-IL-6 antibodies aggravated the disease in terms of histological and functional damage [83]. Anti-IL-6 antibody not only did not improve nephritis but also did not suppress the production of anti-GBM antibody [84].

In summary, IL-6 may be involved in the onset and/or progression of anti-GBM disease, but its role(s) remains unclear. Although only animal models were examined in this review, we found no reports that anti-IL-6 antibodies improved renal lesions, and there were reports that these agents even exacerbated renal lesions, suggesting that anti-IL-6 antibodies may be of little utility.

### 3.7. Complements

Complements are important for identifying and eliminating pathogens [147]. The elimination mechanisms of pathogenic bacteria by complement activation are opsonization, anaphylatoxin (C5a, C3a) production, and membrane attack complex of complements (MAC) formation, among others [147]. Complement activation normally functions as a self-protection against pathogens, but in pathological conditions, it contributes to self-injury [147]. As kidney diseases can be caused by abnormal complement activation, C3 nephropathy and atypical hemolytic uremic syndrome (HUS) are recognized [148]. Moreover, in several glomerular diseases, complements are deposited in glomeruli, and complement activation is involved in the pathogenesis in those diseases.

Eculizumab is a recombinant, humanized monoclonal antibody consisting of a variable region comprised of the complementarity-determining region and the human framework region of mouse anti-human complement C5α chain antibody plus a constant region derived from human IgG [149]. Ravulizumab is constructed with four amino acid substitutions in the eculizumab heavy chain, which destabilize the C5-antibody complex and enhances binding to the Fc receptor, thereby prolonging the half-life of the antibody [150]. Eculizumab has been demonstrated to be effective against paroxysmal nocturnal hemoglobinuria (PNH) [151], atypical HUS [152], myasthenia gravis [153], and neuromyelitis optica spectrum disorder [154], and it has been used clinically. Ravulizumab was also demonstrated to be effective for PNH [155] and atypical HUS [156].

Studies of anti-GBM disease in animal models reached conflicting conclusions on the role played by the complement. Several studies indicated that complements have no effect on nephritis; cobra venom factor (CVF)-induced C3 depletion [157,158] and C5 deficiency [159] had no effect on animal models of anti-GBM disease. Other investigations using an animal model suggested that complements may contribute to the pathogenesis of anti-GBM disease; it was confirmed that the reduction in the severity of nephritis induced with anti-GBM serum was mild in C4-deficient mice [160], moderate in C3-deficient mice [C14], and greater in mice deficient in both C3 and C4 [161]. Moreover, mice with deficient circulating C3 (i.e., C3-deficient mice with C3-sufficient mouse kidney transplants) developed severe glomerular immune complex disease, whereas those with a high level of circulating C3 (C3-sufficient mice that received a transplant of a C3-deficient mouse kidney) had well-preserved glomerular structure and function [162]. Although the glomerular injury by anti-GBM serum in C3-deficient mice was reduced [160,162,163], the glomerular injury in C1q- or C4-deficient mice was enhanced [163]. It has, thus, been suggested that the development of anti-GBM disease may involve an alternative complement pathway rather than the classical pathway or the lectin pathway.

In most patients with anti-GBM disease, a linear binding of C3 to glomerular capillary walls is detected with direct immunofluorescence [164]. Urinary complement levels (C3, C4, C5a, and MAC) were shown to be increased in most of the examined patients with anti-GBM disease (92%, 100%, 100%, and 92%, respectively) [165]. Moreover, urinary C5a values were positively correlated with the serum creatinine at presentation and the percentage of crescents in glomeruli [165]. In another investigation, the patients with anti-GBM disease who had low levels of serum C3 had a higher proportion of glomerular sclerosis progressing to kidney failure compared to the patients with normal levels; in addition, the serum C3 level at diagnosis was an independent protective factor for kidney outcomes of anti-GBM disease [166].

Regarding experimental anti-GBM disease, we found no published report of anti-complement monoclonal antibodies. There are two case reports of the addition of eculizumab to combination therapy with corticosteroids, cyclophosphamide, and plasmapheresis in patients with anti-GBM disease [85,86]. The addition of eculizumab to the treatment of patients with both anti-GBM disease and severe pulmonary disease requiring extracorporeal membrane oxygenation (ECMO) resulted in the successful resolution of the pulmonary disease [85]. In another report, the addition of eculizumab to combination treatment with corticosteroid, cyclophosphamide, plasma exchange, and rituximab improved kidney function in two patients with anti-GBM disease [86]. One of the three patients [86] experienced neutropenia and dermatomal shingles, which were thought to be due to rituximab concomitant use, but there were no clear adverse events associated with eculizumab. However, no clinical studies (RCT, pilot study, or cohort study) involving the anti-complement monoclonal antibodies were identified in our literature search.

In summary, although complements may contribute to the pathogenesis of anti-GBM disease, and although eculizumab was useful for the treatment of anti-GBM disease in a few case reports, there are no reports of this use in animal models or clinical studies. Further investigation is required regarding the efficacy of anti-complement antibodies.

## 4. Conclusions

This article has reviewed immunobiological agents, but other agents useful in anti-GBM disease may also be tried, such as mycophenolic acid [167]/mycophenolate mofetil [168] as an alternative to cyclophosphamide, and imlifidase (the IgG-degrading enzyme of *Streptococcus pyogenes*), instead of plasma exchange therapy [169,170].

**Figure 1:**
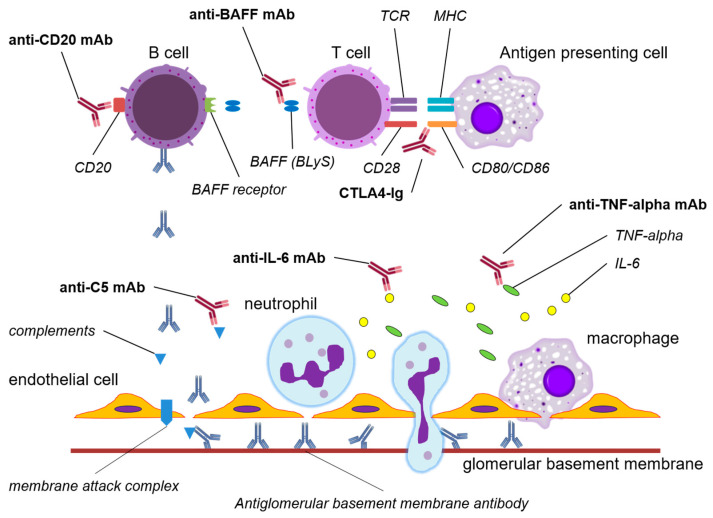
The mechanisms of action of potential immunobiological agents in anti-GBM disease. Abbreviations: TCR, T cell receptor; MHC, major histocompatibility complex; BAFF, B cell activating factor; BLyS, B lymphocyte stimulator; CTLA4, cytotoxic T lymphocyte-associated protein 4; Ig, immunoglobulin; TNF, tumor necrotizing factor; IL-6, interleukin-6; mAb, monoclonal antibody.

Although several immunobiological agents have been used in attempts to treat anti-GBM disease (Figure 1) and used primarily as an alternative to cyclophosphamide, evidence of the usefulness of these agents is still inadequate. Among the several immunobiological agents, only rituximab has been shown to be useful in many case reports or small uncontrolled studies. However, due to the rarity and severity of anti-GBM disease, no randomized clinical trial has been conducted to verify the usefulness of rituximab, and this agent is currently used only in limited cases for patients who are resistant to standard treatments.

## Figures and Tables

**Table 1:** Characteristics and clinical features of patients with anti-GBM disease treated with rituximab.

Characteristics	
Age (years)	43.7 (*n* = 63, range 2–91)
Gender, male/female	34/29
Kidney involvement (%)	61/62 (98.4)
Pulmonary involvement (%)	26/53 (49.1)
**Clinical features at initial presentation**	
Serum creatinine level (mg/dL)	6.95 (*n* = 59, range 0.34–46.6)
End-stage kidney disease (%)	30/49 (61.2)
Positivity of serum anti-GBM antibody (%)	56/61 (91.8)
Serum anti-GBM antibody level (U/mL)	267.8 (*n* = 47, range 0–3060)
Performed renal biopsy (%)	51/57 (89.5)
More than 50% crescentic glomeruli (%)	28/39 (71.8)
Percentage of crescent formation (%)	67.5 (*n* = 39, range 3–100)
**Other treatments**	
Oral corticosteroid (%)	62/64 (96.9)
Initial dose of corticosteroid (mg/day)	52.6 (*n* = 58, range 0–100)
Intravenous corticosteroid therapy (%)	48/56 (85.7)
Dose of intravenous corticosteroid (mg/day)	679.8 (*n* = 53, range 0–1000)
Times of intravenous corticosteroid	2.8 (*n* = 51, range 0–6)
Oral cyclophosphamide (%)	25/58 (43.1)
Intravenous cyclophosphamide (%)	18/54 (33.3)
Plasma exchange (%)	54/62 (87.1)
Times of plasma exchange	12.7 (*n* = 50, 0–100)

Abbreviations: GBM, glomerular basement membrane; *n*, number of patients.

**Table 2:** Treatment methods and outcomes of patients with anti-GBM disease treated with rituximab.

Authors [ref.]	Indication of RTX	From Onset to RTX	RTX Treatments		Follow-up	Outcome	
			Dose	Times		Death	ESKD
Arzoo K, et al. [36]	refractory	1 years	375 mg/m^2^	6	10 mo.	alive	no
Wechsler E, et al. [37]	others	NA	375 mg/m^2^	4	16 mo.	alive	no
Sauter M, et al. [38]	refractory and relapse	6 years	375 mg/m^2^	3	6 yrs.	alive	HD
Schless B, et al. [39]	refractory	9 d	1000 mg/m^2^	1	6 wks.	alive	HD
	refractory	2 wks.	1000 mg/m^2^	1	3 mo.	alive	no
Abenza-Abildua MJ, et al. [40]	tolerance	NA	NA	NA	NA	NA	NA
Shah Y, et al. [41]	tolerance	NA	375 mg/m^2^	4	49 mo.	alive	HD
	tolerance	7 d	375 mg/m^2^	4	37 mo.	alive	no
	NA	NA	375 mg/m^2^	2	33 mo.	alive	no
Vega-Cabrera C, et al. [42]	tolerance	50 d	375 mg/m^2^	4	14 mo.	alive	PD
Syeda UA, et al. [43]	tolerance	28 d	375 mg/m^2^	4	24 mo.	alive	HD
Bandak G, et al. [44]	refractory	2 mo.	1000 mg	1	6 mo.	alive	no
Narayanan M, et al. [45]	tolerance	NA	NA	2	4 mo.	alive	HD
Gray PE, et al. [46]	NA	NA	NA	NA	NA	alive	no
Huang J, et al. [47]	refractory	3 wks.	700 mg	NA	4.5 mo.	alive	no
Calabro N, et al. [48]	tolerance	9 wks.	375 mg/m^2^	8	6 mo.	alive	no
Teixeira AC, et al. [49]	refractory	6 mo.	375 mg/m^2^	4	6 mo.	alive	no
Lemahieu W, et al. [50]	NA	5 d	1000 mg	2	6 mo.	alive	no
Jain R, et al. [51]	refractory	1 mo.	1000 mg	2	2 yrs.	alive	HD
Lester R, et al. [52]	NA	NA	NA	NA	NA	alive	no
Sprenger-Mähr H, et al. [53]	others	2 wks.	1000 mg	2	25 wks.	alive	no
Sporinova B, et al. [54]	refractory	NA	NA	NA	NA	alive	HD
Mannemuddhu SS, et al. [55]	NA	NA	750 mg/m^2^	2	NA	death	HD
Hanna A, et al. [56]	tolerance	NA	1000 mg	NA	2 mo.	alive	NA
Uematsu-Uchida M, et al. [57]	refractory	2 wks.	375 mg/m^2^	2	23 wks.	alive	HD
Isobe S, et al. [58]	NA	NA	200 mg	1	15 mo.	alive	no
Helander L, et al. [59]	NA	7 d	NA	4	17 mo.	alive	PD
Winkler A, et al. [60]	tolerance	NA	1000 mg	2	NA	alive	HD
Povey J, et al. [61]	relapse	2 wks.	1000 mg	2	12 mo.	alive	no
Jen KY, et al. [62]	others	NA	375 mg/m^2^	4	15 mo.	alive	no
Al-Chalabi S, et al. [63]	tolerance	90 d	1000 mg	2	5 mo.	alive	no
Zhang M, et al. [64]	relapse	3 d	500 mg	NA	28 wks.	alive	no
Goda S, et al. [65]	NA	8 d	375 mg/m^2^	NA	45 d	alive	no
Qu W, et al. [66]	NA	NA	200 mg/m^2^	1	5 mo.	alive	no
McAllister J, et al. [67]	tolerance	NA	NA	1	1 yr.	alive	HD
Kanaoka K, et al. [68]	relapse	41 d	375 mg/m^2^	4	2 mo.	alive	no
Honda N, et al. [69]	tolerance	10 d	375 mg/m^2^	4	1 yr.	alive	HD
Touzot M, et al. [70]	6 refractory, 2 relapse	2 mo (0.5–36)	375 mg/m^2^	4	28.9 ± 31.8 mo. (3–93)	0 (0%)	2 (25%)
Heitz M, et al. [71]	all 5 initial treatment	0	375 mg/m^2^	4	17.8 ± 13.8 mo. (4–39)	0 (0%)	4 (80%)
Marques C, et al. [72]	NA	NA	NA	NA	NA	NA	NA
Mayer U, et al. [73]	NA	NA	NA	1	NA	0 (0%)	1 (100%)
Ahmad SB, et al. [74]	NA	NA	NA	NA	NA	0 (0%)	1 (50%)
Yang XF, et al. [75]	4 refractory, 2 relapse, 3 tolerance	198 ± 254 d (30–780)	375 mg/m^2^ (100–600)	3.0 (1–7)	40.6 ± 21.4 mo. (15–184)	0 (0%)	3 (38%)
Jaryal A, Vikrant S. [76]	all 3 initial treatment	0	375 mg/m^2^	4	90 mo.	0 (0%)	1 (33%)

Abbreviations: GBM, glomerular basement membrane; RTX, rituximab; ESKD, end-stage kidney disease; yrs., years; mo., months; wks., weeks; d, days; NA, not available; HD, hemodialysis; PD, peritoneal dialysis. The upper part of this table has described case reports [36,37,38,39,40,41,42,43,44,45,46,47,48,49,50,51,52,53,54,55,56,57,58,59,60,61,62,63,64,65,66,67,68,69], and the lower part has performed cohort studies [70,71,72,73,74,75,76].

## Data Availability

Not applicable.

## Supplementary Materials

The following supporting information can be downloaded at: https://www.mdpi.com/article/10.3390/medicina59112014/s1, Table S1: Prognosis of anti-GBM antibody disease; Table S2: Details of the search strategy used in this review; Table S3: Case reports of patients with anti-glomerular basement membrane disease treated with rituximab; Table S4: Cohort studies of patients with anti-GBM disease treated with rituximab.
