# Supplementary material for: The Immunobiological Agents for Treatment of Antiglomerular Basement Membrane Disease
Source: Medicina (Kaunas). 2023 Nov 16;59(11):2014. doi: 10.3390/medicina59112014 (PMC10673378; doi:10.3390/medicina59112014)
Supplement: Supplementary file 1 [file medicina-59-02014-s001.zip › suppl file 1.pdf]

Suppl. Table S1. Prognosis of anti–GBM antibody disease.

Authors [ref. No.]	Year	N	age, mean (median)	s-Cr levels (mg/dL)	Treatments: immunosuppressant	Treatments: PLEX (%)	Survival rate (%): patients	Survival rate (%): renal
Benoit FL, et al. [7]	1963	52	N.A.	N.A.	none	0	4	2
Proskey AJ, et al. [8]	1970	56	(26.8)	N.A.	various	0	77	23
Wilson CB, et al. [9]	1973	53	28.5	N.A.	various	0	53	13
Beirne GJ, et al. [10]	1977	29	N.A.	N.A.	various	0	42	17
Briggs WA, et al. [11]	1979	7	21.0	6.4 ± 3.1	none or OCS+CY	0	86	0
		7	21.0	3.1 ± 2.9	various	0	71	29
		4	21.0	9.5 ± 13.8	various	100	100	50
Peters DK, et al. [12]	1982	24	N.A.	> 6.8	various	46	79	4
		17	N.A.	< 6.8			94	88
Simpson IJ, et al. [13]	1982	8	28.1	8.0 ± 6.7	none	0	63	25
		4	22.3	4.1 ± 4.0	OCS+AZA	0	100	50
		8	22.1	3.9 ± 3.1	OCS+CY	100	100	63
Johonson JP, et al. [14]	1985	9	22.9	5.3 ± 1.6	OCS+CY	0	89	22
		8	24.8	4.3 ± 1.6		100	100	75
Savage COS, et al. [15]	1986	108	47.8	N.A.	various	42	78	22
Williams PS, et al. [16]	1988	10	57.2	11.8 ± 4.6	various	60	90	10
Herody M, et al. [17]	1993	29	35.2	N.A.	OCS+CY+AZA	0	93	41
Merkel F, et al. [18]	1994	35	34.7	11.4 ± 5.6	OCS+CY	71	89	29
Daly C, et al. [19]	1996	40	[18–76]	5.1 ± 6.8	various	58	98	20
Levy JB, et al. [20]	2001	19	(40) [17-76]	< 5.7	OCS+CY	100	100	95
		13		> 5.7			85	69
		39		ESKD			67	5
Li FK, et al. [21]	2004	10	58.6	7.0 ± 6.4	various	80	70	15
Cui Z, et al. [22]	2005	23	38	< 6.8	various	45	N.A.	61
		46		> 6.8			N.A.	2
Lazor R, et al. [23]	2007	10	(23) [17-65]	< 1.4	OCS+CY	54	N.A.	0
		12		AKI		100	N.A.	83
Hirayama K, et al. [24]	2008	12	48.3	< 6.0	various	50	75	42
		29	52.1	> 6.0		48	79	14
Cui Z, et al. [25]	2011	221	38.5	9.5 ± 5.2	OCS+CY	43	68	19
Taylor DM, et al.[26]	2012	23	45	5.4	OCS+CY	74	78	35
Prabhakar D, et al. [27]	2017	17	39.1	8.6 ± 5.3	various	88	94	41
van Daalen EE, et al. [28]	2018	44	53	4.6	various	88	86	32
Marques C, et al. [29]	2019	119	54	7.2	various	82	92	54
Vavilapalli S, et al. [30]	2020	16	38.7	11.5 ± 6.7	OCS+CY	100	31	6
Zahir Z, et al. [31]	2021	48	46.7	8.4	various	54	71	50

Abbreviations: N, number of patients; AH, alveolar hemorrhage; s-Cr, serum creatinine; ESKD, end-stage kidney disease; AKI, acute kidney injury; OCS, oral corticosteroids; CY, cyclophosphamide; AZA, azathioprine; PLEX, plasma exchange; N.A., not available.
