# Supplementary material for: The Immunobiological Agents for Treatment of Antiglomerular Basement Membrane Disease
Source: Medicina (Kaunas). 2023 Nov 16;59(11):2014. doi: 10.3390/medicina59112014 (PMC10673378; doi:10.3390/medicina59112014)
Supplement: Supplementary file 1 [file medicina-59-02014-s001.zip › suppl. file 2.pdf]

Suppl. Table S2. Details of the search strategy used in this review.

Biologics	The search formula
rituximab	● antiglomerular[All Fields] AND ("basement membrane"[MeSH Terms] OR ("basement"[All Fields] AND "membrane"[All Fields]) OR "basement membrane"[All Fields]) AND ("disease"[MeSH Terms] OR "disease"[All Fields]) AND ("rituximab"[MeSH Terms] OR "rituximab"[All Fields])<br>● goodpasture[All Fields] AND ("rituximab"[MeSH Terms] OR "rituximab"[All Fields])<br>● antiglomerular[All Fields] AND ("basement membrane"[MeSH Terms] OR ("basement"[All Fields] AND "membrane"[All Fields]) OR "basement membrane"[All Fields]) AND anti-CD20[All Fields]<br>● goodpasture[All Fields] AND anti-CD20[All Fields]
anti-BAFF (BLyS) monoclonal antibody	● antiglomerular[All Fields] AND ("basement membrane"[MeSH Terms] OR ("basement"[All Fields] AND "membrane"[All Fields]) OR "basement membrane"[All Fields]) AND ("disease"[MeSH Terms] OR "disease"[All Fields]) AND ("belimumab"[Supplementary Concept] OR "belimumab"[All Fields])<br>● goodpasture[All Fields] AND ("belimumab"[Supplementary Concept] OR "belimumab"[All Fields])<br>● antiglomerular[All Fields] AND ("basement membrane"[MeSH Terms] OR ("basement"[All Fields] AND "membrane"[All Fields]) OR "basement membrane"[All Fields]) AND ("disease"[MeSH Terms] OR "disease"[All Fields]) AND ("TACI receptor-IgG Fc fragment fusion protein"[Supplementary Concept] OR "TACI receptor-IgG Fc fragment fusion protein"[All Fields] OR "atacicept"[All Fields])<br>● goodpasture[All Fields] AND ("TACI receptor-IgG Fc fragment fusion protein"[Supplementary Concept] OR "TACI receptor-IgG Fc fragment fusion protein"[All Fields] OR "atacicept"[All Fields])<br>● antiglomerular[All Fields] AND ("basement membrane"[MeSH Terms] OR ("basement"[All Fields] AND "membrane"[All Fields]) OR "basement membrane"[All Fields]) AND ("disease"[MeSH Terms] OR "disease"[All Fields]) AND ("tabalumab"[Supplementary Concept] OR "tabalumab"[All Fields])<br>● goodpasture[All Fields] AND ("tabalumab"[Supplementary Concept] OR "tabalumab"[All Fields])<br>● antiglomerular[All Fields] AND ("basement membrane"[MeSH Terms] OR ("basement"[All Fields] AND "membrane"[All Fields]) OR "basement membrane"[All Fields]) AND ("disease"[MeSH Terms] OR "disease"[All Fields]) AND ("AMG623 peptibody"[Supplementary Concept] OR "AMG623 peptibody"[All Fields] OR "blisibimod"[All Fields])<br>● goodpasture[All Fields] AND ("AMG623 peptibody"[Supplementary Concept] OR "AMG623 peptibody"[All Fields] OR "blisibimod"[All Fields])<br>● antiglomerular[All Fields] AND ("basement membrane"[MeSH Terms] OR ("basement"[All Fields] AND "membrane"[All Fields]) OR "basement membrane"[All Fields]) AND ("disease"[MeSH Terms] OR "disease"[All Fields]) AND BAFF[All Fields]) OR BLyS[All Fields]<br>● goodpasture[All Fields] AND BAFF[All Fields]) OR BLyS[All Fields]
anti-TNF-alpha monoclonal antibodies	● antiglomerular[All Fields] AND ("basement membrane"[MeSH Terms] OR ("basement"[All Fields] AND "membrane"[All Fields]) OR "basement membrane"[All Fields]) AND ("disease"[MeSH Terms] OR "disease"[All Fields]) AND ("infliximab"[MeSH Terms] OR "infliximab"[All Fields])

	● goodpasture[All Fields] AND ("infliximab"[MeSH Terms] OR "infliximab"[All Fields])<br>● antiglomerular[All Fields] AND ("basement membrane"[MeSH Terms] OR ("basement"[All Fields] AND "membrane"[All Fields]) OR "basement membrane"[All Fields]) AND ("disease"[MeSH Terms] OR "disease"[All Fields]) AND ("adalimumab"[MeSH Terms] OR "adalimumab"[All Fields])<br>● goodpasture[All Fields] AND ("adalimumab"[MeSH Terms] OR "adalimumab"[All Fields])<br>● antiglomerular[All Fields] AND ("basement membrane"[MeSH Terms] OR ("basement"[All Fields] AND "membrane"[All Fields]) OR "basement membrane"[All Fields]) AND ("disease"[MeSH Terms] OR "disease"[All Fields]) AND ("golimumab"[Supplementary Concept] OR "golimumab"[All Fields])<br>● goodpasture[All Fields] AND ("golimumab"[Supplementary Concept] OR "golimumab"[All Fields])<br>● antiglomerular[All Fields] AND ("basement membrane"[MeSH Terms] OR ("basement"[All Fields] AND "membrane"[All Fields]) OR "basement membrane"[All Fields]) AND ("disease"[MeSH Terms] OR "disease"[All Fields]) AND ("certolizumab pegol"[MeSH Terms] OR ("certolizumab"[All Fields] AND "pegol"[All Fields]) OR "certolizumab pegol"[All Fields] OR "certolizumab"[All Fields])<br>● goodpasture[All Fields] AND ("certolizumab pegol"[MeSH Terms] OR ("certolizumab"[All Fields] AND "pegol"[All Fields]) OR "certolizumab pegol"[All Fields] OR "certolizumab"[All Fields])<br>● antiglomerular[All Fields] AND ("basement membrane"[MeSH Terms] OR ("basement"[All Fields] AND "membrane"[All Fields]) OR "basement membrane"[All Fields]) AND ("disease"[MeSH Terms] OR "disease"[All Fields]) AND ("tumor necrosis factor-alpha"[MeSH Terms] OR ("tumor"[All Fields] AND "necrosis"[All Fields] AND "factor-alpha"[All Fields]) OR "tumor necrosis factor-alpha"[All Fields] OR ("tnf"[All Fields] AND "alpha"[All Fields]) OR "tnf alpha"[All Fields]) AND inhibitor[All Fields]<br>● goodpasture[All Fields] AND ("tumor necrosis factor-alpha"[MeSH Terms] OR ("tumor"[All Fields] AND "necrosis"[All Fields] AND "factor-alpha"[All Fields]) OR "tumor necrosis factor-alpha"[All Fields] OR ("tnf"[All Fields] AND "alpha"[All Fields]) OR "tnf alpha"[All Fields]) AND inhibitor[All Fields]
etanercept	● antiglomerular[All Fields] AND ("basement membrane"[MeSH Terms] OR ("basement"[All Fields] AND "membrane"[All Fields]) OR "basement membrane"[All Fields]) AND ("disease"[MeSH Terms] OR "disease"[All Fields]) AND ("etanercept"[MeSH Terms] OR "etanercept"[All Fields])<br>● goodpasture[All Fields] AND ("etanercept"[MeSH Terms] OR "etanercept"[All Fields])
abatacept	● antiglomerular[All Fields] AND ("basement membrane"[MeSH Terms] OR ("basement"[All Fields] AND "membrane"[All Fields]) OR "basement membrane"[All Fields]) AND ("disease"[MeSH Terms] OR "disease"[All Fields]) AND ("abatacept"[MeSH Terms] OR "abatacept"[All Fields])<br>● goodpasture[All Fields] AND ("abatacept"[MeSH Terms] OR "abatacept"[All Fields])<br>● antiglomerular[All Fields] AND ("basement membrane"[MeSH Terms] OR ("basement"[All Fields] AND "membrane"[All Fields]) OR "basement membrane"[All Fields]) AND ("disease"[MeSH Terms] OR "disease"[All Fields]) AND ("cd28 antigens"[MeSH Terms] OR ("cd28"[All Fields]

	AND "antigens"[All Fields]) OR "cd28 antigens"[All Fields] OR "cd28"[All Fields])<br>● goodpasture[All Fields] AND ("cd28 antigens"[MeSH Terms] OR ("cd28"[All Fields] AND "antigens"[All Fields]) OR "cd28 antigens"[All Fields] OR "cd28"[All Fields])<br>● antiglomerular[All Fields] AND ("basement membrane"[MeSH Terms] OR ("basement"[All Fields] AND "membrane"[All Fields]) OR "basement membrane"[All Fields]) AND ("disease"[MeSH Terms] OR "disease"[All Fields]) AND ("ctla-4 antigen"[MeSH Terms] OR ("ctla-4"[All Fields] AND "antigen"[All Fields]) OR "ctla-4 antigen"[All Fields] OR "ctla 4"[All Fields])<br>● goodpasture[All Fields] AND ("ctla-4 antigen"[MeSH Terms] OR ("ctla-4"[All Fields] AND "antigen"[All Fields]) OR "ctla-4 antigen"[All Fields] OR "ctla 4"[All Fields])<br>● antiglomerular[All Fields] AND ("basement membrane"[MeSH Terms] OR ("basement"[All Fields] AND "membrane"[All Fields]) OR "basement membrane"[All Fields]) AND ("disease"[MeSH Terms] OR "disease"[All Fields]) AND ("b7-1 antigen"[MeSH Terms] OR ("b7-1"[All Fields] AND "antigen"[All Fields]) OR "b7-1 antigen"[All Fields] OR "cd80"[All Fields])<br>● goodpasture[All Fields] AND ("b7-1 antigen"[MeSH Terms] OR ("b7-1"[All Fields] AND "antigen"[All Fields]) OR "b7-1 antigen"[All Fields] OR "cd80"[All Fields])<br>● antiglomerular[All Fields] AND ("basement membrane"[MeSH Terms] OR ("basement"[All Fields] AND "membrane"[All Fields]) OR "basement membrane"[All Fields]) AND ("disease"[MeSH Terms] OR "disease"[All Fields]) AND ("b7-2 antigen"[MeSH Terms] OR ("b7-2"[All Fields] AND "antigen"[All Fields]) OR "b7-2 antigen"[All Fields] OR "cd86"[All Fields])<br>● goodpasture[All Fields] AND ("b7-2 antigen"[MeSH Terms] OR ("b7-2"[All Fields] AND "antigen"[All Fields]) OR "b7-2 antigen"[All Fields] OR "cd86"[All Fields])
anti-IL-6 monoclonal antibodies	● antiglomerular[All Fields] AND ("basement membrane"[MeSH Terms] OR ("basement"[All Fields] AND "membrane"[All Fields]) OR "basement membrane"[All Fields]) AND ("disease"[MeSH Terms] OR "disease"[All Fields]) AND ("tocilizumab"[Supplementary Concept] OR "tocilizumab"[All Fields])<br>● goodpasture[All Fields] AND ("tocilizumab"[Supplementary Concept] OR "tocilizumab"[All Fields])<br>● antiglomerular[All Fields] AND ("basement membrane"[MeSH Terms] OR ("basement"[All Fields] AND "membrane"[All Fields]) OR "basement membrane"[All Fields]) AND ("disease"[MeSH Terms] OR "disease"[All Fields]) AND ("sarilumab"[Supplementary Concept] OR "sarilumab"[All Fields])<br>● goodpasture[All Fields] AND ("sarilumab"[Supplementary Concept] OR "sarilumab"[All Fields])<br>● antiglomerular[All Fields] AND ("basement membrane"[MeSH Terms] OR ("basement"[All Fields] AND "membrane"[All Fields]) OR "basement membrane"[All Fields]) AND ("disease"[MeSH Terms] OR "disease"[All Fields]) AND ("interleukin-6"[MeSH Terms] OR "interleukin-6"[All Fields] OR "il 6"[All Fields])<br>● goodpasture[All Fields] AND ("interleukin-6"[MeSH Terms] OR "interleukin-6"[All Fields] OR "il 6"[All Fields])
anti-C5	● antiglomerular[All Fields] AND ("basement membrane"[MeSH Terms]

monoclonal antibodies	OR ("basement"[All Fields] AND "membrane"[All Fields]) OR "basement membrane"[All Fields]) AND ("disease"[MeSH Terms] OR "disease"[All Fields]) AND ("eculizumab"[Supplementary Concept] OR "eculizumab"[All Fields])<br>● goodpasture[All Fields] AND ("eculizumab"[Supplementary Concept] OR "eculizumab"[All Fields])<br>● antiglomerular[All Fields] AND ("basement membrane"[MeSH Terms] OR ("basement"[All Fields] AND "membrane"[All Fields]) OR "basement membrane"[All Fields]) AND ("disease"[MeSH Terms] OR "disease"[All Fields]) AND ("ravulizumab"[Supplementary Concept] OR "ravulizumab"[All Fields])<br>● goodpasture[All Fields] AND ("ravulizumab"[Supplementary Concept] OR "ravulizumab"[All Fields])<br>● antiglomerular[All Fields] AND ("basement membrane"[MeSH Terms] OR ("basement"[All Fields] AND "membrane"[All Fields]) OR "basement membrane"[All Fields]) AND ("disease"[MeSH Terms] OR "disease"[All Fields]) AND C5[All Fields]<br>● goodpasture[All Fields] AND C5[All Fields]
