# Supplementary material for: The Immunobiological Agents for Treatment of Antiglomerular Basement Membrane Disease
Source: Medicina (Kaunas). 2023 Nov 16;59(11):2014. doi: 10.3390/medicina59112014 (PMC10673378; doi:10.3390/medicina59112014)
Supplement: Supplementary file 1 [file medicina-59-02014-s001.zip › suppl. file 3.pdf]

Ref. No	Authors	Year	Age (years old)	Gender	lung involvement	kidney involvement	renal biopsy	Percentage of crescent	complication	at diagnosis	
										A-GBM positivity	A-GBM titer (U/mL)
36	Arzoo K, et al.	2002	73	F	present	NA	NA	NA	HT, HL, IHD, OA	positive	108
37	Wechsler E, et al.	2008	55	M	absent	present	done	10%	HIV, DM, HT	positive	8.6
38	Sauter M, et al.	2009	29	M	present	present	done	NA	NA	positive	1933
39	Schless B, et al.	2009	18	F	NA	present	done	100%	NA	positive	57
39	Schless B, et al.	2009	20	M	NA	present	done	100%	NA	positive	94
40	Abenza-Abildua MJ, et al.	2009	27	M	NA	present	NA	NA	HT	positive	NA
41	Shah Y, et al.	2012	54	M	present	present	NA	NA	HT, multiple sclerosis	positive	NA
41	Shah Y, et al.	2012	64	M	NA	present	done	75%	NA	positive	49
41	Shah Y, et al.	2012	17	M	present	present	done	80%	atypical pneumonia	positive	131
42	Vega-Cabrera C, et al.	2013	27	M	present	present	done	95%	NA	positive	1222
43	Syeda UA, et al.	2013	68	F	absent	present	done	100%	coronary artery disease	positive	NA
44	Bandak G, et al.	2014	24	M	NA	present	done	54%	NA	positive	161
45	Narayanan M, et al.	2014	21	M	present	present	done	100%	NA	positive	191
46	Gray PE, et al.	2015	9	M	NA	present	NA	NA	cerebral vasculitis in X-linked lymphoproliferative disease	NA	NA
47	Huang J, et al.	2016	84	M	present	present	done	NA	pulmonary fibrosis, anterosuperior mediastinal mass, diabetes	positive	NA
48	Calabro N, et al.	2018	50	M	absent	present	done	6%	HIV, KS(Kaposi sarcoma), MCD, HHV-8 infection	positive	4.2
49	Teixeira AC, et al.	2018	24	M	absent	present	done	30%	NA	negative	0
50	Lemahieu W, et al.	2018	29	M	absent	present	done	100%	NA	positive	>680
51	Jain R, et al.	2018	35	F	none	present	done	76%	asthma, rheumatoid arthritis, alopecia totalis, uveitis	positive	185
52	Lester R, et al.	2019	54	F	NA	NA	NA	NA	NA	NA	NA

Ref. No	Authors	Year	Age (years old)	Gender	lung involvement	kidney involvement	renal biopsy	Percentage of crescent	complication	at diagnosis	
										A-GBM positivity	A-GBM titer (U/mL)
53	Sprenger-Mähr H, et al.	2019	30	F	present	present	done	18%	pregnancy	negative	0
54	Sporinova B, et al.	2019	24	M	present	present	done	70%	hypertension, hypothyroidism, morbid obesity	negative	0
55	Mannemuddhu SS, et al.	2019	15	F	NA	present	done	50%	common variable immunodeficiency	positive	194
56	Hanna A, et al.	2019	70	M	present	present	done	40%	prostate cancer, GERD, gout, hypothyroidism, vitiligo, HT,	negative	0
57	Uematsu-Uchida M, et al.	2019	53	F	absent	present	done	54%	no	positive	1170
58	Isobe S, et al.	2020	64	M	absent	present	done	NA	NA	negative	0
59	Helander L, et al.	2021	2	F	NA	present	done	45%	NA	positive	6.3
60	Winkler A, et al.	2021	31	F	present	present	done	100%	NA	positive	137
61	Povey J, et al.	2021	18	F	present	present	done	55%	no	positive	39
62	Jen KY, et al.	2021	4	M	absent	present	done	3%	NA	positive	6.3
63	Al-Chalabi S, et al.	2021	55	M	absent	present	done	50%	psoriatic arthropathy	positive	370.1
64	Zhang M, et al.	2021	41	F	NA	present	done	NA	IgA nephropathy	positive	47.23
65	Goda S, et al.	2022	34	F	present	absent	NA	NA	NA	positive	6.7
66	Qu W, et al.	2022	48	F	absent	present	done	100%	HT	positive	551
67	McAllister J, et al.	2022	14	M	present	present	NA	NA	TTP	positive	NA
68	Kanaoka K, et al.	2022	91	M	present	present	not done	-	atrial fibrillation	positive	4.9
69	Honda N, et al.	2022	68	M	present	present	not done	-	TTP	positive	3060

Ref. No	at diagnosis		indication of rituximab	Use of rituximab	from onset to rituximab	at rituximab treatement			treatments				
	s-Cr (mg/dL)	dialysis dependent				s-Cr (mg/dL)	dialysis dependent	A-GBM titer (U/mL)	rituximab dose	rituximab times	intravenous corticosteroids	daily dose	times
36	NA	NA	refractory	second-line	1 year	NA	NA	51.0	375 mg/m2	6	NA	NA	NA
37	3.5	NA	others	initial	NA	NA	NA	NA	375 mg/m2	4	no	-	-
38	13.3	done	refractory, relapse	second-line	6 years	NA	NA	NA	375 mg/m2	3	done	1000 mg	3
39	2.3	NA	refractory	second-line	9 days	5.96	done	NA	1000 mg/m2	1	no	-	-
39	4.19	no	refractory	second-line	2 weeks	NA	done	NA	1000 mg/m2	1	done	1000 mg	3
40	8.5	done	tolerance	second-line	NA	NA	NA	NA	NA	NA	no	-	-
41	21.19	done	tolerance	second-line	NA	NA	done	NA	375 mg/m2	4	done	1000 mg	4
41	6.06	done	tolerance	second-line	7 days	NA	done	NA	375 mg/m2	4	NA	NA	NA
41	3.08	NA	NA	second-line	NA	NA	NA	NA	375 mg/m2	2	done	1000 mg	4
42	17.1	done	tolerance	second-line	50 days	NA	done	145.0	375 mg/m2	4	done	1000 mg	3
43	11.3	done	tolerance	second-line	28 days	NA	done	NA	375 mg/m2	4	done	1000 mg	3
44	2.8	NA	refractory	second-line	2 months	4.10	not done	59.0	1000 mg	1	done	1000 mg	3
45	12.8	done	tolerance	second-line	NA	NA	done	15.6	NA	2	done	1000 mg	3
46	NA	NA	NA	second-line	NA	NA	NA	NA	NA	NA	NA	NA	NA
47	1.1	NA	refractory	second-line	3 weeks	NA	NA	NA	700 mg/week	NA	NA	NA	NA
48	3.7	no	tolerance	second-line	9 weeks	6.80	done	NA	375 mg/m2	8	done	1000 mg	6
49	1.7	no	refractory	second-line	6 months	1.34	not done	NA	375 mg/m2	4	done	1000 mg	3
50	20	done	NA	second-line	5 days	NA	done	NA	1000 mg	2	done	1000 mg	NA
51	8.07	done	refractory	second-line	1 month	NA	done	85.0	1000 mg	2	done	1000 mg	3
52	NA	NA	NA	second-line	NA	NA	NA	NA	NA	NA	NA	NA	NA

Ref. No	at diagnosis		indication of rituximab	Use of rituximab	from onset to rituximab	at rituximab treatement			treatments				
	s-Cr (mg/dL)	dialysis dependent				s-Cr (mg/dL)	dialysis dependent	A-GBM titer (U/mL)	rituximab		intravenous corticosteroids		
									dose	times		daily dose	times
53	0.4	no	others	second-line	2 weeks	NA	not done	NA	1000 mg	2	no	-	-
54	12.05	done	refractory	second-line	NA	NA	done	NA	NA	NA	done	500 mg	NA
55	5.5	done	NA	second-line	NA	NA	NA	NA	750 mg/m2	2	done	10 mg/kg	3
56	NA	NA	tolerance	second-line	NA	NA	NA	0.0	1000 mg	NA	done	1000 mg	3
57	8.86	done	refractory	second-line	2 weeks	NA	done	317.0	375 mg/m2	2	done	500 mg	3
58	1.11	no	NA	second-line	NA	NA	NA	NA	200 mg	1	done	500 mg	3
59	5.27	done	NA	second-line	7 days	NA	done	NA	NA	4	done	30 mg/kg	3
60	4.75	NA	tolerance	second-line	NA	NA	NA	20.0	1000 mg	2	NA	NA	NA
61	2.4	no	relapse	second-line	2 weeks	3.46	no	5.2	1000 mg	2	done	NA	NA
62	0.34	no	others	second-line	NA	NA	no	NA	375 mg/m2	4	done	30 mg/kg	3
63	4.7	no	tolerance	second-line	90 days	NA	NA	NA	1000 mg	2	done	250 mg	NA
64	1.78	NA	relapse	second-line	3 days	NA	NA	NA	500 mg	NA	done	1000 mg	3
65	0.53	NA	NA	second-line	8 days	NA	NA	NA	375 mg/m2	NA	done	1000 mg	3
66	1.74	NA	NA	second-line	NA	NA	NA	NA	200 mg/m2	1	done	500 mg	3
67	46.6	done	tolerance	second-line	NA	NA	done	NA	NA	1	done	NA	NA
68	1.08	NA	relapse	second-line	41 days	NA	NA	NA	375 mg/m2	4	done	1000 mg	3
69	30.7	done	tolerance	second-line	10 days	NA	NA	NA	375 mg/m2	4	done	1000 mg	3

Ref. No	treatments									follow-up	outcome		at the end of follow-up	
	oral corticosteroid		cyclophosphamide					plasma exchange					s-Cr	A-GBM titer
		dose	poCYC	CYC dose	IVCY	IVCY dose	IVCY times	done	times		Death	ESKD	(mg/dL)	(U/mL)
36	done	NA	done	NA	NA		NA	done	NA	10 mo	alive	no	NA	< 3
37	done	40 mg	none	-	none	-	-	none	NA	16 mo	alive	no	1.20	0
38	done	1 mg/kg	done	100 mg	none		-	done	NA	6 yrs	alive	HD	-	NA
39	done	100 mg	done	200 mg	none		-	done	NA	6 wks	alive	HD	-	0
39	done	100 mg	done	200 mg	none		none	done	NA	3 mo	alive	no	3.39	0
40	done	30 mg	done	150 mg	none		-	none	-	NA			NA	NA
41	no	NA	done		IVCY	500 mg	NA	done	50	49 mo	alive	HD	-	0
41	done	NA	done	500 mg	NA		7 days	NA	NA	37 mo	alive	no	2.94	< 6
41	done	30 mg	none	none	none		none	done	17	33 mo	alive	no	1.13	0
42	done	1 mg/kg	done	2 mg/kg	done	1000 mg	1	done	18	14 mo	alive	PD	-	0
43	done	1 mg/kg	done	1 - 2 mg/kg	none		none	done	NA	24 mo	alive	HD	-	0
44	done	60 mg	done	NA	none	-	none	done	100	6 mo	alive	no	2.06	16
45	done	80 mg	none	-	none	-	-	done	NA	4 mo	alive	HD	-	3
46	done	NA	done	NA	NA		NA	NA	NA	NA	alive	no	NA	0
47	done	80 mg	none	-	done	400 mg	3 weeks	done	6	4.5 mo	alive	no	1.39	0
48	done	60 mg	done	1 mg/kg	none	-	-	done	NA	6 mo	alive	no	2.20	0
49	done	1 mg/kg	done	3 mg/kg/day	none	-	none	none	-	6 mo	alive	no	1.36	NA
50	done	1 mg/kg	none	-	none	-	-	done	10	6 mo	alive	no	2.40	1.7
51	done	60 mg	done	100 mg	none	-	none	done	10	2 yrs	alive	HD	-	0
52	done	NA	done	NA	NA		NA	done	NA	NA	alive	no	NA	NA

Ref. No	treatments									follow-up	outcome		at the end of follow-up	
	oral corticosteroid		cyclophosphamide					plasma exchange					s-Cr	A-GBM titer
		dose	poCYC	CYC dose	IVCY	IVCY dose	IVCY times	done	times		Death	ESKD	(mg/dL)	(U/mL)
53	done	1 mg/kg	none	-	done	855 mg	1	done	5	25 wks	alive	no	NA	NA
54	done	60 mg	none	-	done	1200 mg	-	done	NA	NA	alive	HD	-	NA
55	done	60 mg	none	-	done	10 mg/kg	2	done	10	NA	death	HD	-	0
56	done	60 mg	none	-	none	-	-	done	6	2 mo	alive	NA	NA	0
57	done	50 mg	NA	NA	NA	NA	NA	done	9	23 wks	alive	HD	-	22.6
58	done	5 mg	NA	NA	NA	NA	NA	done	3	15 mo	alive	no	1.00	NA
59	done	2 mg/kg	none	NA	done	NA	2	done	15	17 mo	alive	PD	-	0
60	done	60 mg	none	-	IVCY	1000mg	1	done	14	NA	alive	HD	-	0
61	done	60 mg	done	NA	oral		-	done	21	12 mo	alive	no	0.66	< 0.8
62	done	2 mg/kg	NA	-	-		-	none	-	15 mo	alive	no	NA	0
63	done	30 mg	done	2 mg/kg	done	1000 mg	2	done	NA	5 mo	alive	no	NA	0
64	done	40 mg	NA	NA	NA	NA	NA	done	6	28 wks	alive	no	1.33	0
65	done	60 mg	NA	NA	NA	NA	NA	done	4	45 days	alive	no	NA	NA
66	done	50 mg	done	1 mg/kg	done		1	done	4	5 mo	alive	no	NA	0
67	done	NA	NA	NA	done	500 - 750 mg/m2	4	done	33	1 yr	alive	HD	NA	0.8
68	done	60 mg	done	50 mg	none		-	done	13	2 mo	alive	no	NA	0
69	done	1 mg/kg	none	-	-		-	done	NA	1 yr	alive	HD	NA	0
