# Supplementary material for: The Immunobiological Agents for Treatment of Antiglomerular Basement Membrane Disease
Source: Medicina (Kaunas). 2023 Nov 16;59(11):2014. doi: 10.3390/medicina59112014 (PMC10673378; doi:10.3390/medicina59112014)
Supplement: Supplementary file 1 [file medicina-59-02014-s001.zip › suppl. file 4.pdf]

Ref. No	Authors	Year	No. of patients	Age (years old)	Male Gender	lung involvement	kidney involvement	renal biopsy	Percentage of crescent	complication	at diagnosis	
											A-GBM positivity	A-GBM titer (U/mL)
70	Touzot M, et al.	2015	8	21<br>(16 - 65)	2<br>(25%)	5<br>(63%)	8<br>(100%)	8<br>(100%)	NA	NA	8<br>(100%)	69.5<br>(3.5 - 148)
71	Heitz M, et al.	2018	5	72<br>(17 - 96)	1<br>(20%)	3<br>(60%)	5<br>(100%)	4<br>(80%)	70%<br>(20 - 100)	hypertension 2<br>diabetes 0	5<br>(100%)	NA
72	Marques C, et al.	2019	11	NA	NA	NA	NA	NA	NA	NA	NA	NA
73	Mayer U, et al.	2020	1	NA	NA	NA	1<br>(100%)	1<br>(100%)	100%	NA	NA	NA
74	Ahmad SB, et al.	2021	2	70.0<br>(59 - 81)	1<br>(50%)	0<br>(0%)	2<br>(100%)	2<br>(100%)	61.5%<br>(23 - 100)	NA	2<br>(100%)	180
75	Yang XF, et al.	2022	8	55.5 ± 21.0<br>(16 - 82)	5<br>(63%)	1<br>(13%)	8<br>(100%)	6<br>(75%)	85.3%<br>(32.0 - 95.8)	pneumonia 3<br>diabetes 0	8<br>(100%)	(46 - >200)
76	Jaryal A, Vikrant S.	2022	3	46.3 ± 12.1<br>(37 - 60)	2<br>(67%)	0<br>(0%)	3<br>(100%)	3<br>(100%)	82.8 ± 19.0	diabets 67%<br>UTI 67%	3<br>(100%)	NA

Ref. No	at diagnosis		indication of rituximab				Use of rituximab			at rituximab treatement		
	s-Cr (mg/dL)	dialysis dependent	refractory	relapse	tolerance	others	initial	second-line	from initial to rituximab	s-Cr (mg/dL)	dialysis dependent	A-GBM titer (U/mL)
70	3.19 (0.74 - 4.79)	4 (50%)	6 (75%)	2 (25%)	0 (0%)	0 (0%)	0 (0%)	8 (100%)	2 mo (0.5 - 36 mo)	1.78 ± 0.90 (0.70 - 3.60)	1 (13%)	23 (8 - 40)
71	5.50 ± 3.57 (0.53 - 9.05)	4 (80%)	0 (0%)	0 (0%)	0 (0%)	5 (100%)	5 (100%)	0 (0%)	0	5.50 ± 3.57 (0.53 - 9.05)	4 (80%)	NA *
72	NA	NA	NA	NA	NA	NA	NA	NA	NA	NA	NA	NA
73	eGFR 8	1 (100%)	NA	NA	NA	NA	NA	NA	NA	NA	NA	NA
74	17.8 (10.6 - 25.02)	2 (100%)	NA	NA	NA	NA	NA	NA	NA	NA	NA	NA
75	2.78 (1.03 - 9.62)	3 (38%)	4 (50%)	2 (25%)	3 (38%)	0 (0%)	2 (25%)	6 (75%)	198 ± 254 (30 - 780)	NA	3 (38%)	NA
76	6.2 ± 2.5	2 (67%)	0 (0%)	0 (0%)	0 (0%)	3 (100%)	3 (100%)	0 (0%)	0	6.2±2.5	67%	NA

Ref. No	treatments													
	rituximab		intravenous corticosteroids			oral corticosteroid		cyclophosphamide					plasma exchange	
	dose	times	yes	dose (mg/day)	times	yes	dose	poCYC	CYC dose	IVCY	IVCY dose	IVCY times	yes	times
70	375 mg/m2/w	4	6 (75%)	NA	NA	7 (88%)	NA	0 (0%)	-	8 (100%)	NA	3 (1 - 9)	5 (63%)	13 (9 - 25)
71	375 mg/m2/w	4	3 (60%)	373 (120 - 500)	3.3 (3 - 4)	5 (100%)	1 mg/kg	0 (0%)	-	0 (0%)	-	-	5 (100%)	16.6 ± 6.6 (9 - 23)
72	NA	NA	NA	NA	NA	NA	NA	NA	NA	NA	NA	NA	NA	NA
73	NA	1	1 (100%)	NA	5	1 (100%)	50 mg	1 (100%)	NA	NA	NA	NA	1 (100%)	6
74	NA	NA	NA	NA	NA	1 (50%)	NA	0 (0%)	-	0 (0%)	-	-	1 (50%)	NA
75	375 mg/m2/w (100 - 600)	3.0 (1 - 7)	8 (100%)	(160 - 1000)	(2 - 6)	8 (100%)	(20 - 60)	5 (63%)	50 mg	0 (0%)	-	-	8 (100%)	12.3 ± 5.2 (3 - 17)
76	375 mg/m2/w	4	3 (100%)	500	3	3 (100%)	NA	0 (0%)	-	0 (0%)	-	-	3 (100%)	7

Ref. No	follow-up (months)	outcome Death	ESKD	at the end of follow-up s-Cr (mg/dL)	A-GBM titer (U/mL)	adverse effect
70	28.9 ± 31.8 (3 - 93)	0 (0%)	2 (25%)	NA	0	NA
71	17.8 ± 13.8 (4 - 39)	0 (0%)	4 (80%)	NA	NA	2 infection
72	NA	NA	NA	NA	NA	NA
73	NA	0 (0%)	1 (100%)	NA	NA	NA
74	NA	0 (0%)	1 (50%)	NA	NA	NA
75	40.6 ± 21.4 (15 - 184)	0 (0%)	3 (38%)	2.25 ± 1.38 (1.23 - 4.52)	0	NA
76	90	0 (0%)	1 (33%)	NA	NA	NA
